# scGR-seq: Integrated analysis of glycan and RNA in single cells

**DOI:** 10.1016/j.xpro.2022.101179

**Published:** 2022-02-18

**Authors:** Haruki Odaka, Haruka Ozaki, Hiroaki Tateno

**Affiliations:** 1 Cellular and Molecular Biotechnology Research Institute, National Institute of Advanced Industrial Science and Technology (AIST), Tsukuba Central 6, 1-1-1 Higashi, Tsukuba, Ibaraki 305-8566, Japan; 2 Bioinformatics Laboratory, Faculty of Medicine, University of Tsukuba, 1-1-1 Tennodai, Tsukuba, Ibaraki 305-8575, Japan; 3 JST PRESTO, Tsukuba Central 6, 1-1-1 Higashi, Tsukuba, Ibaraki 305-8566, Japan

**Keywords:** Cell Biology, Cell culture, Single Cell, Genomics, Sequencing, RNAseq

## Abstract

Glycans are structurally diverse molecules found on the surface of living cells. The protocol details a system developed for combined analysis of glycan and RNA in single cells (scGR-seq) using human induced pluripotent stem cells (hiPSCs) and hiPSC-derived neural progenitor cells (NPCs). scGR-seq consists of DNA-barcoded lectin-based glycan profiling by sequencing (scGlycan-seq) and single-cell transcriptome profiling (scRNA-seq). scGR-seq will be an essential technique to delineate the cellular heterogeneity of glycans across multicellular systems.

For complete details on the use and execution of this profile, please refer to Minoshima et al. (2021).

## Highlights

• Protocols for combined analysis of glycan and RNA in single cells (scGR-seq)
• scGR-seq is a DNA-barcoded lectin-based glycan profiling
• scGR-seq analysis of hiPSCs and NPCs were shown as an example
• scGR-seq is adaptable for any cells, tissues, and organs across organisms

## Graphical abstract

**Figure:**
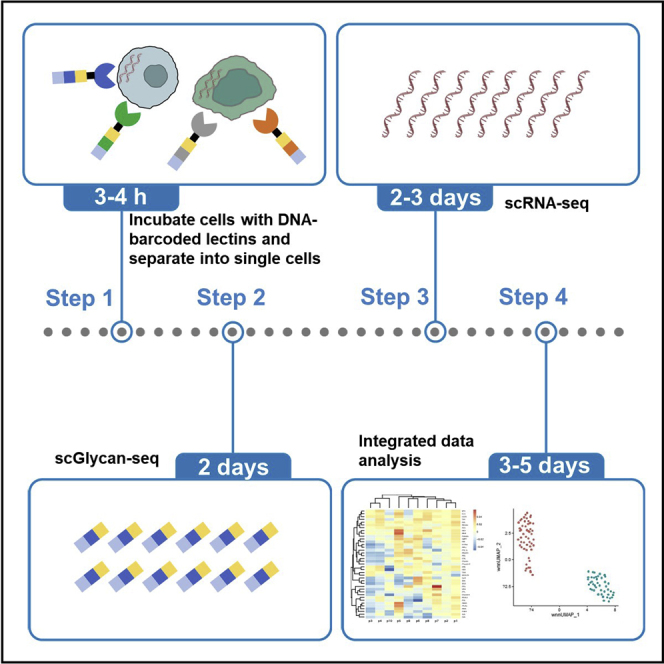

## Before you begin

The protocol below describes the specific steps for using human induced pluripotent stem cells (hiPSCs) and hiPSC-derived neural progenitor cells (NPCs). However, we have also used this protocol in other cells, such as human dermal fibroblasts, hiPSC-derived neurons, and several cell lines.

**Figure 1:**
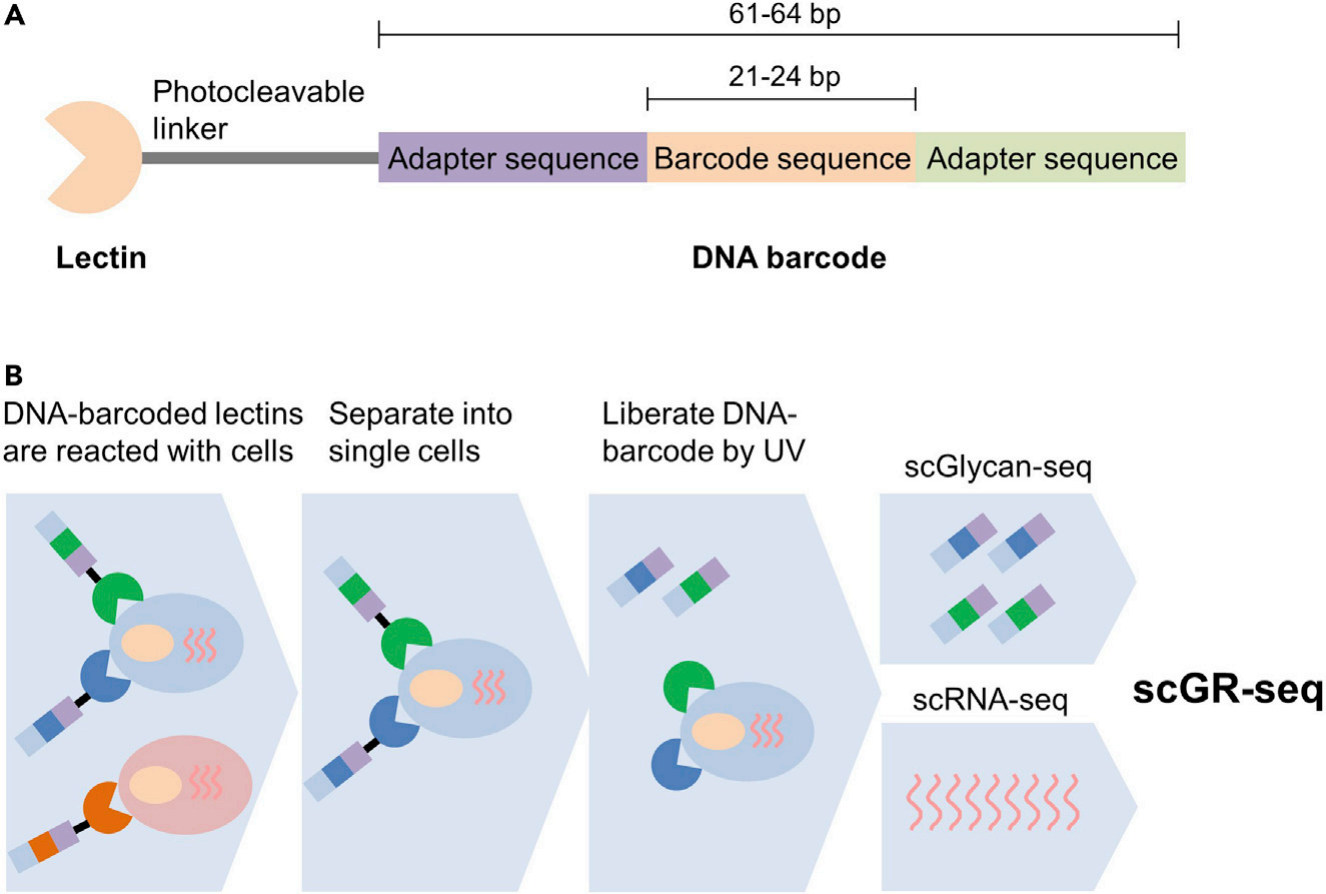
Schematic diagram of scGlycan/RNA sequencing (A) Illustration of the DNA-barcoded lectin. (B) Schematic illustration of scGlycan-seq, scRNA-seq, and scGR-seq. Figure reprinted with permission from Minoshima et al. (2021).

This protocol consists of three major steps (Figure 1)-

(i) Single cell Glycan-seq (scGlycan-seq)

(ii) Single cell RNA-seq (scRNA-seq)

(iii) Integrated data analysis (scGR-seq)

Standard cell culture procedures and humidified incubators are required for the maintenance of cell culture.

For scRNA-seq, we use RamDA-seq, a plate-based single-cell total RNA sequencing method (Hayashi et al., 2018).

We use the next-generation sequencer, MiSeq (Illumina) and the barcode DNA counting system (Minoshima et al., 2021) to count DNA-barcodes derived from each lectin.

### Preparation of sugar-immobilized column

**Timing: 2 days**

1. Wash 20 g of Sepharose CL-4B gel using a glass filter in 500 mL of Milli-Q water.
2. Suspend the Sepharose CL-4B gel in 30 mL of Milli-Q water and add 3 mL of epichlorohydrin and 13 mL of 2 N NaOH followed by incubation at 40°C for 2 h with shaking.
3. Wash the Sepharose CL-4B with 500 mL of Milli-Q water.
4. Dissolve 10 mmol of sugar ligands for lectins (see Table 1) in 80 mL of 0.1 M NaOH (pH 13.0) and mix all of it with epoxy-activated Sepharose CL-4B (prepared in step 3). Incubate at 40°C for 24 h followed by washing with 500 mL of Milli-Q water.

**Table 1:** Lectin lists

	Name	Species (origin)	Rough specificity	Oligo DNA sequence	Sugar-immobilized column used for purification	Eluents used for purification
1	rPVL	*Psathyrella velutina*	Sia, GlcNAc	CGACGCTCTTCCGATCTCTGTCGCC CTGAACAACGGCAATGGCAGTCAG ATCGGAAGAGCACAC	GlcNAc	0.2 M GlcNAc
2	SNA	*Sambucus nigra*	α2-6Sia	CGACGCTCTTCCGATCTCTGTGCCG CTAGCCAGGGTTGGACTGTCAGAT CGGAAGAGCACAC	Lac	0.2 M Lac
3	SSA	*Sambucus sieboldiana*	α2-6Sia	CGACGCTCTTCCGATCTCTGGCACT CGTTGCTGGGTCTGGGGACGTCAG ATCGGAAGAGCACAC	Lac	0.2 M Lac
4	TJAI	*Trichosanthes japonica*	α2-6Sia	CGACGCTCTTCCGATCTCTGACAG CTACTCGTGCGGGAAAGCAGTCAG ATCGGAAGAGCACAC	Lac	0.2 M Lac
5	rPSL1a	*Polyporus squamosus*	α2-6Sia	CGACGCTCTTCCGATCTCTGGGTC TGGGGTCAACTCCGTGGCGTGTC AGATCGGAAGAGCACAC	Lac	0.2 M Lac
6	rDiscoidinII	*Dictyostelium dicodeum*	LacNAc, Galβ1-3GalNAc (T), aGalNAc (Tn)	CGACGCTCTTCCGATCTCTGGGCG AAGTCTCAATCGGCGATCGGGTC AGATCGGAAGAGCACAC	Lac	0.2 M Lac
7	rCGL2	*Coprinopsis cinerea*	GalNAcα1-3Gal (A), PolyLacNAc	CGACGCTCTTCCGATCTCTGTGTG GCAGCCATTCGTTCCTCCGCGTC AGATCGGAAGAGCACAC	Lac	0.2 M Lac
8	rC14	*Gallus gallus domesticus*	Branched LacNAc	CGACGCTCTTCCGATCTCTGACCC AAGCGATCTGACTGTCCACCGTC AGATCGGAAGAGCACAC	Lac	0.2 M Lac
9	GSLII	*Griffonia simplicifolia*	bisecting GlcNAc	CGACGCTCTTCCGATCTCTGTCCT CCAAGGAGCCGCCACACCGTCA GATCGGAAGAGCACAC	GlcNAc	0.2 M GlcNAc
10	rSRL	*Sclerotium rolfsii*	Core1,3, agalacto N-glycan	CGACGCTCTTCCGATCTCTGCGTG CTGACGATGGGTGGCAGTGTCA GATCGGAAGAGCACAC	GlcNAc	0.2 M GlcNAc
11	rF17AG	*Escherichia coli*	GlcNAc	CGACGCTCTTCCGATCTCTGAGC GGCTGGTGTGTAGGGGCCAGTC AGATCGGAAGAGCACAC	GlcNAc	0.2 M GlcNAc
12	rGRFT	*Griffithsia sp.*	Man	CGACGCTCTTCCGATCTCTGCGT ATGGCGGTAGCGGTGGTAGCGT CAGATCGGAAGAGCACAC	Man	0.2 M Man
13	ConA	*Canavalia ensiformis*	Man	CGACGCTCTTCCGATCTCTGTGG GAGTCCACAGGGAAGCAGTGTG TCAGATCGGAAGAGCACAC	Man	0.2 M Me-α-Man
14	rOrysata	*Oryza sativa*	Manα1-3Man, High-man, biantenna	CGACGCTCTTCCGATCTCTGGGG TGGCAATGGAGGCAGTGCACAG TCAGATCGGAAGAGCACAC	Man	0.2 M Man
15	rPALa	*Phlebodium aureum*	Man5, biantenna	CGACGCTCTTCCGATCTCTGTGGT GAACGGCCTGCAAGTCGTGTGTC AGATCGGAAGAGCACAC	Man	0.2 M Man
16	rBanana	*Musa acuminata*	Manα1-2Manα1-3(6)Man	CGACGCTCTTCCGATCTCTGGTGG GAATGGTGGCTCAGCGTTCGTCA GATCGGAAGAGCACAC	Man	0.2 M Man
17	rCalsepa	*Calystegia sepium*	Biantenna with bisecting GlcNAc	CGACGCTCTTCCGATCTCTGGGC GGCAACAATCCCATTGCGTGTCA GATCGGAAGAGCACAC	Man	0.2 M Man
18	rRSL	*Ralstonia solanacearum*	αMan, α1-2Fuc (H), α1-3Fuc (Lex), α1-4Fuc (Lea)	CGACGCTCTTCCGATCTCTGTGC GAATGCAGCCAACACGCAGTCA GATCGGAAGAGCACAC	Man	0.2 M Man
19	rBC2LA	*Burkholderia cenocepacia*	αMan, High-man	CGACGCTCTTCCGATCTCTGACT GATGCGCGTTTAGCCCCGAGTC AGATCGGAAGAGCACAC	Man	0.2 M Man
20	rAAL	*Aleuria aurantia*	Fucose	CGACGCTCTTCCGATCTCTGACT GGCAGAAAGGTCGCGAAGAGC GTCAGATCGGAAGAGCACAC	Fuc	0.2 M Fuc
21	rRSIIL	*Ralstonia solanacearum*	αMan, α1-2Fuc (H), α1-3Fuc (Lex), α1-4Fuc (Lea)	CGACGCTCTTCCGATCTCTGTCC GTCCATTCGCGTCTACACCGCG TCAGATCGGAAGAGCACAC	Fuc	0.2 M Fuc
22	rPhoSL	*Pholiota squarrosa*	α1-6Fuc	CGACGCTCTTCCGATCTCTGTGG AAAGTGGGTCGCTCAGTGGGGTC AGATCGGAAGAGCACAC	Fuc	0.2 M Fuc
23	rAOL	*Aspergillus oryzae*	αMan, α1-2Fuc (H), α1-3Fuc (Lex), α1-4Fuc (Lea)	CGACGCTCTTCCGATCTCTGTGG CAAGTCTGCTGGGATCATGGCGT CAGATCGGAAGAGCACAC	Fuc	0.2 M Fuc
24	rBC2LCN	*Burkholderia cenocepacia*	Fucα1-2Galβ1-3GlcNAc (GalNAc)	CGACGCTCTTCCGATCTCTGATG TTGCGAAAGCGGGCATACGGTCA GATCGGAAGAGCACAC	Fuc	0.2 M Fuc
25	UEAI	*Ulex europaeus*	Fucα1-2Galβ1-4GlcNAc	CGACGCTCTTCCGATCTCTGAGT GACGACGGTGGCTTGCCAGTCA GATCGGAAGAGCACAC	Fuc	0.2 M Fuc
26	TJAII	*Trichosanthes japonica*	Fucα1-2Galβ1-4GlcNAc, GalNAcβ1-4GlcNAc	CGACGCTCTTCCGATCTCTGGC CGGCGAAATCACATGTGTTTGC GTCAGATCGGAAGAGCACAC	Fuc	0.2 M Fuc
27	rGC2	*Geodia cydonium*	α1-2Fuc (H), αGalNAc (A), αGal (B)	CGACGCTCTTCCGATCTCTGCTC GCCACGCATCCACTGGTGGTCA GATCGGAAGAGCACAC	Lac	0.1 M Lac
28	rMOA	*Marasmius oreades*	αGal (B)	CGACGCTCTTCCGATCTCTGGG ACTCCGATTGTAGGCTGGCAGT GTCAGATCGGAAGAGCACAC	Melibiose	0.1 M Lac
29	rPAIL	*Pseudomonas aeruginosa*	α,βGal, αGalNAc (Tn)	CGACGCTCTTCCGATCTCTGTGC CACGATGCGTTCTGTGGAGCCGT CAGATCGGAAGAGCACAC	Lac	0.1 M Lac
30	rGal3C	*Homo sapiens*	LacNAc, polylactosamine	CGACGCTCTTCCGATCTCTGTGG CCTTTCACTTCAACCCACGCGTC AGATCGGAAGAGCACAC	Lac	0.1 M Lac
31	rLSLN	*Laetiporus sulphureus*	LacNAc, polylactosamine	CGACGCTCTTCCGATCTCTGCGC TTGCTTGGGTTTGCCAGTCGGTC AGATCGGAAGAGCACAC	Lac	0.1 M Lac
32	HPA	*Helix pomatia*	αGalNAc (A, Tn)	CGACGCTCTTCCGATCTCTGGCG AGTCCGTATTGCCGTCCACCGGT CAGATCGGAAGAGCACAC	GalNAc	0.2 M GalNAc
33	rPPL	*Pleurocybella porrigens*	α,βGalNAc (A, Tn, LacDiNAc)	CGACGCTCTTCCGATCTCTGTGGT ACTCGCACCTTGGAAACCGTGTC AGATCGGAAGAGCACAC	Lac	0.1 M Lac
34	rCNL	*Clitocybe nebularis*	α,βGalNAc (A, Tn, LacDiNAc)	CGACGCTCTTCCGATCTCTGTGGT GCAGCTCTGGTTGGCTCAGTCAG ATCGGAAGAGCACAC	Lac	0.1 M Lac
35	WFA	*Wisteria floribunda*	Terminal GalNAc, LacDiNAc	CGACGCTCTTCCGATCTCTGGCC AAAGCTGCAGATGGCCTTGCCGT CAGATCGGAAGAGCACAC	GalNAc	0.2 M GalNAc
36	rABA	*Agaricus bisporus*	Galβ1-3GalNAc (T), GlcNAc	CGACGCTCTTCCGATCTCTGTGG GTGGCTCTGGGACCTCAGGGTC AGATCGGAAGAGCACAC	GlcNAc	0.2 M GlcNAc
37	rDiscoidinI	*Dictyostelium Discoideum*	Gal	CGACGCTCTTCCGATCTCTGTCCA ACCGCGCAACATCACGACCAGTC AGATCGGAAGAGCACAC	Lac	0.2 M Lac
38	rMalectin	*Homo sapiens*	Glcα1-2Glc	CGACGCTCTTCCGATCTCTGGCCG TGTTGGTCGTGCTTCGGGTCAGAT CGGAAGAGCACAC	Maltose	0.2 M Maltose
39	CSA	*Oncorhynchus keta*	Rhamnose, Galα1-4Gal	CGACGCTCTTCCGATCTCTGAGCG TTCTTCTTGGCACCCGCTGTCAGA TCGGAAGAGCACAC	Rhamnose	0.2 M Rhamnose
40	mIgG	*Mus musculus*	ND	CGACGCTCTTCCGATCTCTGGCTT GGCAAGCGTTCCTGGCTGTCAGA TCGGAAGAGCACAC	Protein G	0.1 HCl
41	gIgG	*Ovis aries*	ND	CGACGCTCTTCCGATCTCTGACG AGCGACTCAAGGACAAGTGGTC AGATCGGAAGAGCACAC	Protein G	0.1 M Glycine-HCl
5. Resuspend the sugar-immobilized Sepharose CL-4B in 50 mL of 1 M monoethanolamine (pH 8.0) and incubate at 37°C overnight to block the excess epoxy groups.
6. Wash the sugar-immobilized Sepharose CL-4B with 0.1 M acetate buffer (pH 4.0)/0.5 M NaCl and 0.1 M Tris-HCl (pH 8.0)/0.5 M NaCl.
7. Resuspend the sugar-immobilized Sepharose CL-4B in Milli-Q water, and store it at 4°C until further use.
8. Pack 1 mL of the sugar-immobilized Sepharose CL-4B into Micro Bio-Spin Chromatography Columns and store it at 4°C until further use.

***Note:*** 1 g of Sepharose gel contains 0.5 mmol of epoxy groups. The amount of gel to prepare can be reduced depending on the purification scale.

**CRITICAL:** Epichlorohydrin is a toxic substance.

### Conjugation of lectin with DNA-barcode

**Timing: 1 day**

9. Mix 100 μL of lectin (1 mg/mL in PBS) with 10× concentration of photocleavable-dibenzylcyclooctyne-N-hydroxysuccinimide ester (PC-DBCO-NHS) and incubate at 20°C for 1 h in the dark.
10. Add 10 μL of 1 M Tris-HCl (pH 8.0), and incubate at 20°C for 15 min in the dark to block excess PC-DBCO-NHS.
11. After incubation, remove the free PC-DBCO-NHS using the G-25 desalting miniature column (see materials and equipment).
12. Add 200 μM of 5′-azide-DNA (10× concentration, see Table 1) to the PC-DBCO-lectin to generate DNA-barcoded lectins.

### Purification of DNA-barcoded lectins

**Timing: 2 days**

**Figure 2:**
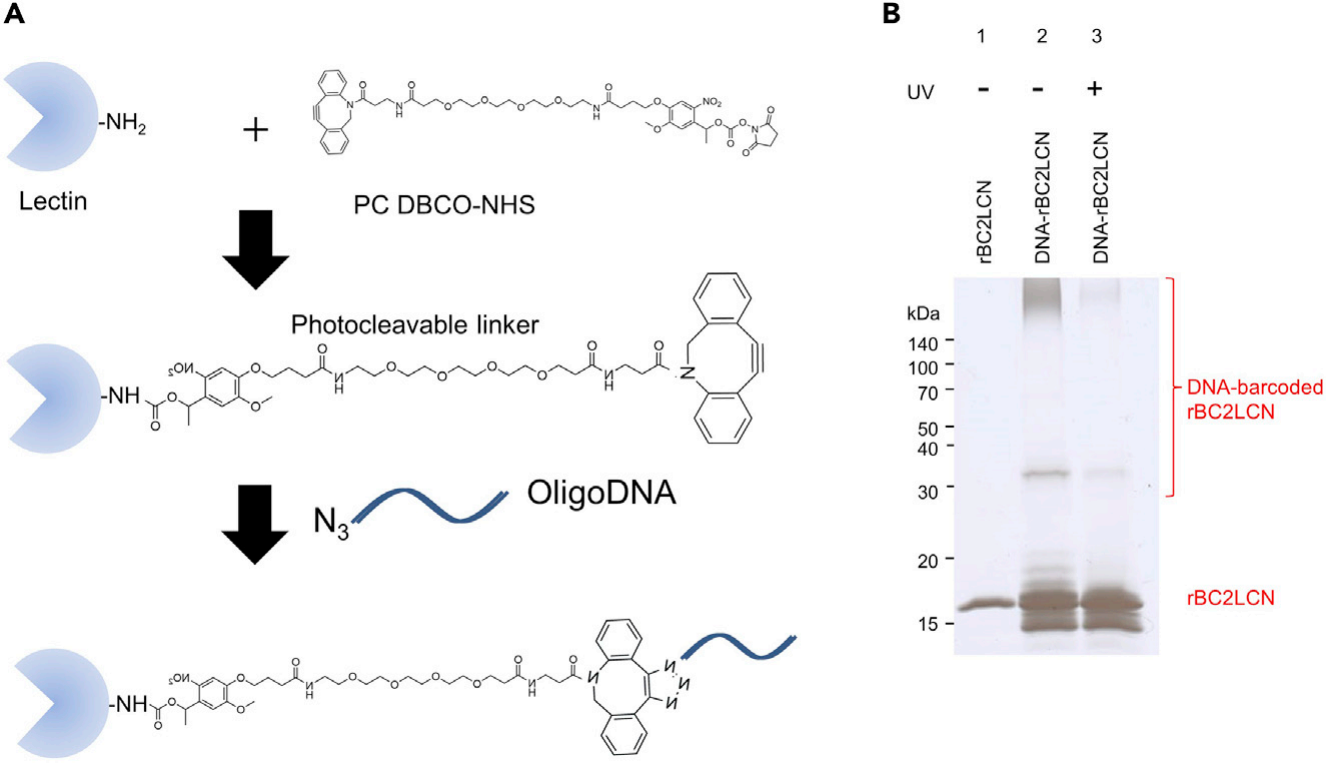
Preparation of DNA-barcoded lectins (A) Illustration of reaction process to conjugate DNA oligonucleotides to lectin. (B) rBC2LCN shows a single band at 16 kDa (lane 1). DNA-barcoded rBC2LCN exhibited a high-molecular weight smear band at >140 kDa (lane 2). Cleavage of DNA barcodes from rBC2LCN by UV exposure collapses the smear to the MW of rBC2LCN (16 kDa) (lane 3). Figure reprinted with permission from Minoshima et al. (2021).
18. Load 8 μL of the samples as well as 5 μL of Prestained Protein Size Marker onto 17% SDSPAGE gel. Run the SDSPAGE using SDS running buffer at 100 V for 20 min.
19. Stain the SDSPAGE gel with GelRed followed by the manufacturer’s protocol. This step can stain free as well as lectin-conjugated DNA-barcodes
20. Stain the SDSPAGE gel with silver staining reagents followed by the manufacturer’s protocol. The gel used for the GelRed staining can be used for silver staining.
21. Recover the elution fractions and dialyze the purified DNA-barcoded lectins against 0.1× PBS (for dialysis) using Tube-O-Dialyzer, Medi 8kD.
22. Concentrate the DNA-barcoded lectins using a centrifugal filter (Amicon ultra 0.5 mL 10K) having a 10 kDa molecular weight cut off.
23. Quantify the protein and DNA concentration using the Bradford and Quant-iT OliGreen ssDNA Reagent Kit, respectively, and determine the DNA-to-lectin ratio.
24. Mix 41 DNA-barcoded probes (5 μg/mL, final concentration, for each lectin) (Table 1) into a 1.5 mL tube and fill up to 100 μL with PBS/BSA.
25. Store it at −30°C.

***Note:*** Any lectins can be used for scGR-seq, but we recommend to check whether the lectins show no reaction each other using assays such as lectin blotting. We labeled 41 probes with DNA barcodes (Table 1), which cover a wide range of glycans such as sialylated, galactosylated, mannosylated, GlcNAcylated, and fucosylated glycans.

13. The sugar-immobilized Sepharose CL-4B column (1 mL in miniature column) is washed with 1 mL PBSE at 4°C.
14. Add 100 μL of PBSE into the DNA-barcoded lectin solutions and apply onto the sugar-immobilized Sepharose CL-4B column. Recover the flow-through fraction (100 μL).
15. Wash the sugar-immobilized Sepharose CL-4B column with 400 μL of PBSE three times. Recover each of the wash fractions (400 μL each).
16. Add 400 μL of the elution solution comprising PBSE containing an appropriate sugar for each lectin (see Table 1). Repeat this step three times. Recover each of the elution fractions (400 μL each).
17. Analyze the DNA-barcoded lectins by SDS-PAGE (Figure 2). Mix 4 μL of each fraction of the purification steps (lectin only, flow-through, wash, elution) with 4 μL of SDS sample buffer.

**Caution**: Some lectins are eluted in washing fractions. In this case, recover the wash fractions and use for the experiments.

## Key resources table

**Table:** 

REAGENT or RESOURCE	SOURCE	IDENTIFIER
**Antibodies**		

PE Mouse anti-Human PAX6	BD Biosciences	clone No. O18-1330
PE Mouse anti-NESTIN	BD Biosciences	clone 25/NESTIN (RUO)
Mouse anti-Oct3/4	Santa Cruz Biotechnology, Inc.	Cat#sc-5279
Mouse anti-SSEA-4	Merck	Cat#MAB4304
Donkey anti-mouse IgG Alexa Fluor 488 polyclonal antibody (pAb)	Thermo Fisher Scientific	Cat#A21202

**Chemicals, peptides, and recombinant proteins**		

PBS	Fujifilm Wako Pure Chemical Co.	Cat#045-29795
EDTA	Sigma-Aldrich	Cat#09-1420-5
BSA	Merck KGaA	Cat#A3059
Tris-HCl	Nacalai Tesque, Inc.	Cat#35434-21
Na_2_HPO_4·_12H_2_O	Fujifilm Wako Pure Chemical Co.	Cat#196-02835
NaCl	Fujifilm Wako Pure Chemical Co.	Cat#191-01665
KCl	Fujifilm Wako Pure Chemical Co.	Cat#163-03545
KH_2_PO_4_	Fujifilm Wako Pure Chemical Co.	Cat#166-04255
Nuclease-free water	QIAGEN	Cat#129114
Epichlorohydrin	NacalaiTesque, Inc.	Cat#14415-05
Monoethanolamine	Cytiva	Cat#BR-1000-50
PC-DBCO-NHS ester	Click Chemistry Tools	Cat#1160-10
WIDE-VIEW Prestained Protein Size Marker	Fujifilm Wako Pure Chemical Co.	Cat#230-02221
GelRed	Biotium	Cat#41002
mTeSR Plus	VERITAS	Cat#ST-100-0276
Matrigel	CORNING	Cat#REF 354230
Gentle Cell Dissociation Reagent	VERITAS	Cat#ST-100-0485
mFreSR Cryopreservation Medium	VERITAS	Cat#ST-05855
MesenPRO RS medium	Thermo Fisher Scientific KK	Cat#12746012
Dibenzocyclooctyne-N-hydroxysuccinimidyl ester	Funakoshi Co., Ltd	Cat#A133-25
Y-27632	Fujifilm Wako Pure Chemical Co.	Cat#3924591
Accutase	Innovative Cell Technologies, Inc.	Cat#AT104
DMEM/F12 media	Thermo Fisher Scientific KK	Cat#11330032
CaCl_2_	Fujifilm Wako Pure Chemical Co.	Cat#039-00475
PhiX	Illumina KK	Cat#FC-110-3001
Rnase Away	Thermo Fisher Scientific KK	Cat#7002
rPVL	Tateno et al. (2011)	N/A
SNA	VECTOR LABORATORIES	Cat#L-1300
SSA	Seikagaku	Cat#300177
TJAI	Seikagaku	Cat#300186
rPSL1a	Tokyo Chemical Industry Co. Ltd.	Cat#R0225
rDiscoidinII	Tateno et al. (2011)	N/A
rCGL2	Fujifilm Wako Pure Chemical Co.	Cat#033-23771
rC14	Tateno et al. (2011)	N/A
GSLII	VECTOR LABORATORIES	Cat#L-1210
rSRL	Tokyo Chemical Industry Co. Ltd.	Cat#R0228
rF17AG	Fujifilm Wako Pure Chemical Co.	Cat#062-06281
rGRFT	Tokyo Chemical Industry Co. Ltd.	Cat#R0229
ConA	Seikagaku	Cat#300036
rOrysata	Fujifilm Wako Pure Chemical Co.	Cat#159-03281
rPALa	Fujifilm Wako Pure Chemical Co.	Cat#164-26731
rBanana	Fujifilm Wako Pure Chemical Co.	Cat#025-18661
rCalsepa	Fujifilm Wako Pure Chemical Co.	Cat#031-23831
rRSL	Tateno et al. (2011)	N/A
rBC2LA	Tateno et al. (2011)	N/A
rAAL	Fujifilm Wako Pure Chemical Co.	Cat#018-25201
rRSIIL	Tateno et al. (2011)	N/A
rPhoSL	Tateno et al. (2011)	N/A
rAOL	Tokyo Chemical Industry Co. Ltd.	Cat#
rBC2LCN	Fujifilm Wako Pure Chemical Co.	Cat#029-18061
UEAI	VECTOR LABORATORIES	Cat#L-1060
TJAII	Seikagaku	Cat#300187
rGC2	Tateno et al. (2011)	N/A
rMOA	Tokyo Chemical Industry Co. Ltd.	Cat#R0227
rPAIL	Fujifilm Wako Pure Chemical Co.	Cat#167-26721
rGal3C	Fujifilm Wako Pure Chemical Co.	Cat#079-06351
rLSLN	Tokyo Chemical Industry Co. Ltd.	Cat#R0226
HPA	EY Laboratories, Inc	Cat#L-3601
rPPL	Fujifilm Wako Pure Chemical Co.	Cat#168-26751
rCNL	Fujifilm Wako Pure Chemical Co.	Cat#039-23631
WFA	VECTOR LABORATORIES	Cat#L-1350
rABA	Fujifilm Wako Pure Chemical Co.	Cat#015-24851
rDiscoidinI	Fujifilm Wako Pure Chemical Co.	Cat#045-33541
rMalectin	Fujifilm Wako Pure Chemical Co.	Cat#062-06281
CSA	J-OIL-MILLS, Inc	Cat#10001005
mIgG	Jackson ImmunoResearch	Cat#015-000-003

**Critical commercial assays**		

Silver Stain MS Kit	Fujifilm Wako Pure Chemical Co.	Cat#299-58901
Bradford protein assay	Bio-Rad Laboratories	Cat#5000001JA
Quant-iT OliGreen ssDNA Reagent	Thermo Fisher Scientific KK	Cat#O7582
PowerUp SYBR Green Master Mix	Thermo Fisher Scientific KK	Cat#A25741
STEMdiff SMADi Neural Induction Kit (STEMdiff™ Neural Induction Medium/SMADi Neural Induction Supplement)	VERITAS	Cat#08581
NEBNext Ultra II Q5 Master Mix	New England BioLabs Japan Inc	Cat#M0544S
Agencourt AMPure XP kit	Beckman Coulter, Inc.	Cat#BC-A63880
Miseq Reagent Kit v2 50 Cycles	Illumina KK	Cat#MS-102-2001
GenNext RamDA-seq Single Cell Kit	TOYOBO	Cat#RMD-101
R-phycoerythrin Labeling Kit	Dojindo Laboratories Co. Ltd.	Cat#LK23
RNeasy Mini Kit	QIAGEN	Cat#74104
DNA Clean & Concentrator Kit-5, with Uncapped Column (200preps)	ZYMO RESEARCH	Cat#D4004
DNA-500 kit	Shimadzu Co.	Cat#S292-27910-91
25 bp DNA ladder	Thermo Fisher Scientific KK	Cat#10597-011
pUC19 DNA/MspI (HpaII) Marker	Thermo Fisher Scientific KK	Cat#SM0221
Nextera XT DNA Library Preparation Kit (96 samples)	Illumina KK	Cat#FC-131-1096
Nextera XT Index Kit v2 Set A (96 indexes, 384 samples)	Illumina KK	Cat#FC-131-2001
Nextera XT Index Kit v2 Set B (96 indexes, 384 samples)	Illumina KK	Cat#FC-131-2002

**Deposited data**		

All raw data of scRNA-seq	Minoshima et al. (2021)	Cat#GSE151642
All raw data of scGlycan-seq	Minoshima et al. (2021)	N/A

**Experimental models: Cell lines**		

Human: iPS cell line 201B7	RIKEN BioResource Center	Cat#HPS0063

**Oligonucleotides**		

Primer: i7 index primer		Table S1
Primer: i5 index primer		Table S1
5′-azide-DNA oligonucleotide		Table 1

**Software and algorithms**		

Barcode DNA counting system	Minoshima et al. (2021)	https://github.com/bioinfo-tsukuba/barcode-dna-counting-system
PoolQ-3.3.2	The Broad Institute Genetic Perturbation Platform	https://portals.broadinstitute.org/gpp/public/software/poolq
Subio Platform version 1.24.5849	Subio Inc.	https://www.subioplatform.com/
R version 3.6.1	The R Foundation	https://www.r-project.org/
Seurat version 4.02	Satija Lab	https://satijalab.org/seurat/index.html

**Other**		

Sepharose CL-4B	Cytiva	Cat#17015001
Protein low binding microtubule	WATSON Co.,Ltd.	Cat#PK-15C-500
Protein low binding microtubule	WATSON Co.,Ltd.	Cat#PK-15C-500
Amicon ultra 0.5 mL 10K	Merck KGaA	Cat#UFC501096
TOPick I Live Cell Pick system	Yodaka Giken	N/A
Glass needle	Yodaka Giken	N/A
Optical Flat 8-Cap Strips for 0.2 mL tube strips	Bio-Rad Laboratories	Cat#TCS0803
DynaMag-2 Magnet (magnetic stand)	Thermo Fisher Scientific KK	Cat#12321D
MiSeq	Illumina, Inc.	N/A
MultiNA	Shimadzu Co.	N/A
UVP Blak-Ray XX-15L UV Bench Lamp	analytik jena US, An Endress+Hauser Company	Cat#95-0042-12
Sephadex G-25 fine	GE Healthcare	Cat#17-0032-01
Tubu-O-Dialyzer, Medi 8kD	BIOSCIENCES	Cat#786617
PANTERA Gel 17% 20w	DRC	Cat#NSV-3X6P20
Micro Bio-Spin Chromatography Columns	Bio-Rad Laboratories	Cat#7326204

## Materials and equipment

**Table:** 0.1 M acetate buffer (pH 4.0)/0.5 M NaCl

Reagent	Final concentration	Amount
Sodium acetate trihydrate	0.1 M	13.6 g
NaCl	0.5 M	29.22 g

***Note:*** Dissolve sodium acetate trihydrate and NaCl with 500 mL MilliQ, adjust the pH to 4.0 by acetic acid, and fill up to 1 L with MilliQ.

**Table:** 0.1 M Tris-HCl (pH 8.0)/0.5 M NaCl

Reagent	Final concentration	Amount
Tris-HCl	0.1 M	12.1 g
NaCl	0.5 M	29.22 g

***Note:*** Dissolve Tris-HCl and NaCl with 500 mL MilliQ, adjust the pH to 4.0 by acetic acid, and fill up to 1 L with Milli Q.

**Table:** SDS sample buffer

Reagent	Final concentration	Amount
1 M Tris-HCl (pH 6.8)	0.125 M	1.25 mL
SDS	4%	4 g
Sucrose	10%	1 g
Bromophenol Blue	0.004%	0.4 mg

***Note:*** Fill up to 10 mL with MilliQ.

**Table:** SDS running buffer

Reagent	Final concentration	Amount
Tris-HCl	25 mM	3.03 g
Glycine	192 mM	14.4 g
SDS	0.1%	1 g

***Note:*** Fill up to 10 mL with MilliQ.

**Table:** PBS (for dialysis)

Reagent	Final concentration	Amount
Na₂HPO₄·12H₂O	5.86 mM	2.1 g
NaCl	130 mM	8 g
KCl	2.7 mM	0.2 g
KH₂PO₄	1.48 mM	0.2 g
EDTA	1 mM	3.72 g

***Note:*** Fill up to 1 L with MilliQ.

**Table:** PBSE

Reagent	Final concentration	Amount
Na₂HPO₄·12H₂O	5.86 mM	2.1 g
NaCl	130 mM	8 g
KCl	2.7 mM	0.2 g
KH₂PO₄	1.48 mM	0.2 g
EDTA	1 mM	3.72 g

***Note:*** Fill up to 1 L with MilliQ.

**Table:** G-25 desalting miniature column

Reagent	Final concentration	Amount
Sephadex G-25 fine	n/a	0.8 mL
Micro Bio-Spin Chromatography Columns	n/a	n/a

***Note:*** Wash Sephadex G-25 fine with TBS, and dispense 0.8 mL of Sephadex G-25 fine into the column and store it at 4°C.

**Table:** PBS/BSA

Reagent	Final concentration	Amount
PBS	n/a	n/a
BSA	1%	10 g
Total	n/a	1 L

***Note:*** Filter the reagent using 0.22 μm PVDF membrane and store it at 4°C.

**Table:** PBS/BSA/CaCl_2_

Reagent	Final concentration	Amount
PBS/BSA	n/a	1 L
CaCl_2_	1 mM	N/A
Total	n/a	1 L

**Table:** STEMdiff™ Neural Induction Medium/SMADi Neural Induction Supplement

Reagent	Final concentration	Amount
STEMdiff™ Neural Induction Medium	n/a	250 mL
STEMdiff™ SMADi Neural Induction Supplement	n/a	0.5 mL
Total	n/a	250.5 mL

***Note:*** Aliquot and store at −20°C; however, it can be stored at 2°C–8°C for up to 2 weeks if not used immediately.

## Step-by-step method details

### Cell culture of hiPSCs

**Timing: 3–4 days**

**CRITICAL:** Perform all cell culture experiments inside a biosafety cabinet, and wear personal protective equipment, including gloves and goggles.

1. Coat each well of 6-well plate with 1 mL of Matrigel and let it sit at room temperature (RT) for 1 h.
2. Thaw the mTeSR Plus media at 37°C for 5–15 min. Plate the appropriate number of 201B7 hiPSCs in a 6-well plate containing 2 mL of the mTeSR Plus media.
3. Culture the cells for 2–3 days in a CO_2_ incubator (with CO_2_ level set to 5%).
4. Recover the cells with gentle cell dissociation reagent and resuspend them in the mTeSR Plus media supplemented with 10 μM Y-27632.

**Pause point:** hiPSC are suspended in mFreSR Cryopreservation Medium and stored in liquid nitrogen.

### Generation of hiPSC-derived neural progenitor cells (NPCs)

**Timing: 11 days**

5. Thaw STEMdiff™ Neural Induction Medium and STEMdiff™ SMADi Neural Induction Supplement at room temperature (15°C–25°C) or overnight (2°C–8°C). Swirl both media thoroughly.
6. Add 0.5 mL of STEMdiff™ SMADi Neural Induction Supplement to 250 mL of STEMdiff™ Neural Induction Medium (NIM). Mix them thoroughly and warm them to room temperature before use.
7. Coat each well of 6-well plate with 1 mL of Matrigel and let it sit at room temperature for 1 h.
8. Wash the hiPSC cultured well with 2 mL of phosphate-buffered saline (PBS).
9. Add 1 mL of gentle cell dissociation reagent and incubate at 37°C for 8–10 min.
10. Pipet up and down 3–5 times to dissociate the cell aggregates. Collect the cells in a 15 mL conical tube.
11. Wash the plate with 2 mL PBS and collect the remaining cells into a 15 mL conical tube.
12. Count the viable cells with a hemocytometer using Trypan blue dye method.
13. Centrifuge the 15 mL conical tube at 300×*g* for 4 min. Aspirate and discard the supernatant without disturbing the cell pellet.
14. Resuspend the cell pellets with NIM supplemented with 10 μM Y-27632 to achieve a final concentration of 1 × 10^6^ cells/mL.
15. Aspirate the Matrigel from a 6-well plate and add 2 mL cell suspension (2 × 10^6^ cells/well) into a single well of Matrigel-coated plate.
16. Incubate the cells at 37°C, 5% CO_2_ for 11 days. Fresh NIM without Y-27632 is used for medium changes every other day.
17. Aspirate the media from the plates, add 1 mL of Accutase per well, and incubate at 37°C, 5% CO_2_ for 5 min.
18. Collect the cell suspension in a 15 mL conical tube. Wash the plates with 2 mL pre-warmed DMEM/F12 media, and collect the residual in the same tube. Centrifuge the cell suspension at 300×*g* for 4 min, and the resulting cell pellet is resuspended in NIM and used for the evaluation of the differentiation state by qRT-PCR and fluorescence staining of hiPSC markers (POU5F1) and NPC markers (SOX1, NESTIN, PAX6, FOXG1).

**Pause point:** hiPSC-derived NPCs are suspended in mFreSR Cryopreservation Medium and stored in liquid nitrogen.

### Single cell glycan-seq

**Timing: 2 days**

**CRITICAL:** Contamination with DNA and DNase will significantly affect the experiments. Perform all experiments inside a biosafety cabinet in the dark, and wear personal protective equipment, including gloves and goggles. Take extreme care while handling all the reagents to prevent contamination with DNA and DNase. Prepare and dispense all reagents on ice unless otherwise stated.

19. Incubate the cells with DNA-barcoded lectins.
  a. Take 1 × 10^5^cells in a 1.5 mL microtube and centrifuge at 600×*g* for 4 min at 4°C.
  b. Discard the supernatant and resuspend the pellet in 1 mL of PBS/BSA. Repeat the washing step twice.
  c. Discard the supernatant and resuspend in 90 μL of PBS/BSA/CaCl_2_ containing 10 μL of DNA-barcoded lectin mix (5 μg/mL of each lectin, 10 μL).
  d. Incubate the cells on ice for 1 h in the dark followed by centrifuge at 600×*g* for 4 min at 4°C.
  e. Discard the supernatant and resuspend the pellet in 1 mL of PBS/BSA/CaCl_2_. Repeat the washing step three times.
  f. Add 200 μL of PBS/BSA/CaCl_2_ and count the cell number.

**CRITICAL:** CaCl_2_ should be added in the solution when you use lectins, which require Calcium for glycan-binding activity.

20. Dispense single-cells into tubes.
  a. Dispense single cells into the cap of Optical Flat 8-Cap Strips (for 0.2 mL tubes) by manually picking them using TOPick I Live Cell Pick system or other methods.
  b. Cover the tubes with the cap containing single cells, spin down, and keep them on ice.
21. Expose the cells to UV light for 15 min using UVP Blak-Ray XX-15L UV Bench Lamp to liberate DNA-barcodes from lectins.
22. Centrifuge at 3,549×*g* for 30 s at 4°C and transfer the supernatant into a new 0.2 mL 8-tube strips, kept on ice.

**CRITICAL:** Be careful not to touch the cells with the pipette tip. You can leave 0.5 μL of supernatant in the tube.

**Pause point:** The supernatant can be stored at −80°C.

23. Add 2.5 μL of cell lysis buffer into the tube and cover the tube with a cap.
24. Spin down and store at −80°C for single-cell RNA-seq (see the next section “single cell RNA-seq.”
25. Perform PCR to amplify the DNA-barcodes for sequencing.
  a. Prepare the PCR mix as follows: each sample contains 25 μL of total reaction volume- 9.75 μL of supernatant (template), 12.5 μL of NEBNext UltraII Q5 Master Mix, 0.25 μL of i5 index primer (100 mM), and 2.5 μL of i7 index primer (10 mM).
  b. Perform the PCR reactions as follows.

**Table:** PCR cycling conditions

Steps	Temperature	Time	Cycles
Initial Denaturation	98°C	45 s	1
Denaturation	98°C	10 s	25–35 cycles
Annealing and extension	65°C	50 s	
Final extension	65°C	5 min	1
Hold	4°C	forever

26. Purify the PCR products.
  a. Combine 16 samples into a 1.5 mL microtube.
  b. Add 320 μL of AMPure into it and gently pipette the contents 10 times.
  c. After incubation at RT for 5 min, expose the tubes to the magnetic stand for 2 min.
  d. Discard the supernatants without disturbing the magnetic beads on the magnetic stand.
  e. Add 1 mL of 80% ethanol into the 1.5 mL microtube followed by incubation at RT for 30 s on the magnetic stand. Discard the supernatant and repeat the washing step three times.
  f. Air-dry the magnetic beads at RT for 10 min.
    **CRITICAL:** When beads are dried, the color of the beads becomes lighter. If it dried too much, it would be difficult to elute.
  g. Remove 1.5 mL microtube from the magnetic stand and add 160 μL of 10 mM Tris (pH 8.5).
  h. Gently pipette the contents 10 times and incubate at RT for 2 min.
  i. Expose the tube to the magnetic stand and carefully collect the supernatant to a 15 mL tube.

27. Concentrate the PCR products.
  a. Add 5 volumes of DNA Binding Buffer to the 15 mL tube and mix briefly by vortexing.
  b. Transfer the mixture into a Collection Tube containing Zymo-Spin Column.
  c. Centrifuge for 30 s and discard the flow-through.
  d. Add 200 μL of DNA Wash Buffer to the column and centrifuge it for 30 s. Repeat the washing step.
  e. Add 20 μL DNA Elution Buffer directly to the column matrix and incubate at RT for 1 min. Transfer the column to a 1.5 mL microcentrifuge tube and centrifuge at 20,400×*g* for 30 s at 4°C to elute the DNA.
28. Use 6.5 μL of the elution fraction to analyze the size and quantity of the PCR products, using the microchip electrophoresis system–MultiNA with DNA-500 kit–according to the manufacturer’s instructions. A single band between 150 and 175 bp will appear if the DNA library is constructed successfully.

**Pause point:** DNA library can be stored at −20°C.

***Alternatives:*** For the analysis of the size and quantity of the PCR products, Agilent Bioanalyzer or Agilent TapeStation could be considered.

29. Denaturing library DNA
  (for > 4 nM of library DNA)
  a. Dilute the concentration of library DNA to 4 nM with nuclease-free water and mix it in equal amounts.
  b. Mix 4 μL of the library DNA mixture of all the samples with 4 μL of 0.1 N NaOH, briefly by vortexing and spin down.
  c. Incubate at RT for 5 min and keep them on ice.
  d. Add 4 μL of 2 nM library DNA to 796 μL of pre-chilled HT1 for a total of 800 μL (10 pM) library DNA. Mix briefly by vortexing and spin down.
    (for < 4 nM of library DNA)
  e. Mix each library DNA in equal amounts
  f. Mix 2 μL of the library DNA mixture of all the samples with 2 μL of 0.1 N NaOH, briefly by vortexing and spin down.
  g. Incubate at RT for 5 min and keep them on ice.
  h. Add 2 μL of 200 mM Tris-HCl (pH 7.0) and mix briefly by vortexing and spin down.
  i. Add 6 μL of library DNA to 534 μL of pre-chilled HT1 for a total of 540 μL, library DNA. Mix briefly by vortexing and spin down.

30. Denaturing PhiX
  a. Mix 1 μL of 10 nM PhiX, 4 μL of nuclease-free water, and 5 μL of 0.1 N NaOH for a total of 10 μL, 1 nM of Phix. Mix briefly by vortexing and spin down.
  b. Incubate at RT for 5 min and chill on ice.
  c. Mix 2 μL of 1 nM denatured PhiX and 248 μL of pre-chilled HT1 for a total of 250 μL (8 pM PhiX). Mix briefly by vortexing and spin down.
31. Mix 540 μL of library DNA (step 29) with 130 μL of 8 pM PhiX (step 30).
32. Heat at 96°C for 2 min and chill on ice immediately followed by incubation for 5 min.
33. Load 600 μL of the library mix into the reagent cartridge of MiSeq Reagent kit and run the setup according to the manufacturer’s instructions.

### Single cell RNA-seq

**Timing: 1–2 days**

**CRITICAL:** Contamination with RNase and DNA will significantly affect the experiments. Perform all experiments inside a biosafety cabinet in the dark, and wear personal protective equipment, including gloves, masks, and goggles. Wipe all instruments used for the experiment, i.e., pipette, centrifuge, mixer, thermal cycler, laboratory bench, with RNase remover –RnaseAway. Take extreme care while handling all the reagents to prevent contamination with RNase. In order to inactivate RNase and maintain enzyme activity, prepare and dispense all reagents on ice, unless otherwise stated.

34. Prepare cDNA library using a full-length total RNA-sequencing method –Random displacement amplification sequencing (RamDA-seq)–from single cells, according to manufacturer’s instructions.
35. Quantify the library DNA from individual samples derived from single cells, using the microchip electrophoresis system–MultiNA with DNA-12000 kit–according to the manufacturer’s instructions. A band of 150–600 bp will appear if the DNA library is constructed successfully.
36. Pool and mix each library DNA and transfer 50–100 fmol into a 1.5 mL tube.
37. Sequence the mixed library DNA using a next-generation sequencer such as Novaseq6000 according to the sequencer guidelines.

## Expected outcomes

**Figure 3:**
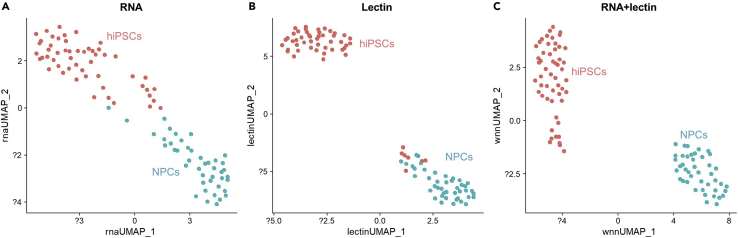
UMAP visualization for hiPSCs and NPCs (A–C) UMAP plot based on (A) only the scRNA-seq data and (B) only the scGlycan-seq, and (C) both scRNA-seq and scGlycan-seq (scGR-seq) data of hiPSCs (n = 53, red) and NPCs (n = 43, green). Figure reprinted with permission from Minoshima et al. (2021).

**Figure 4:**
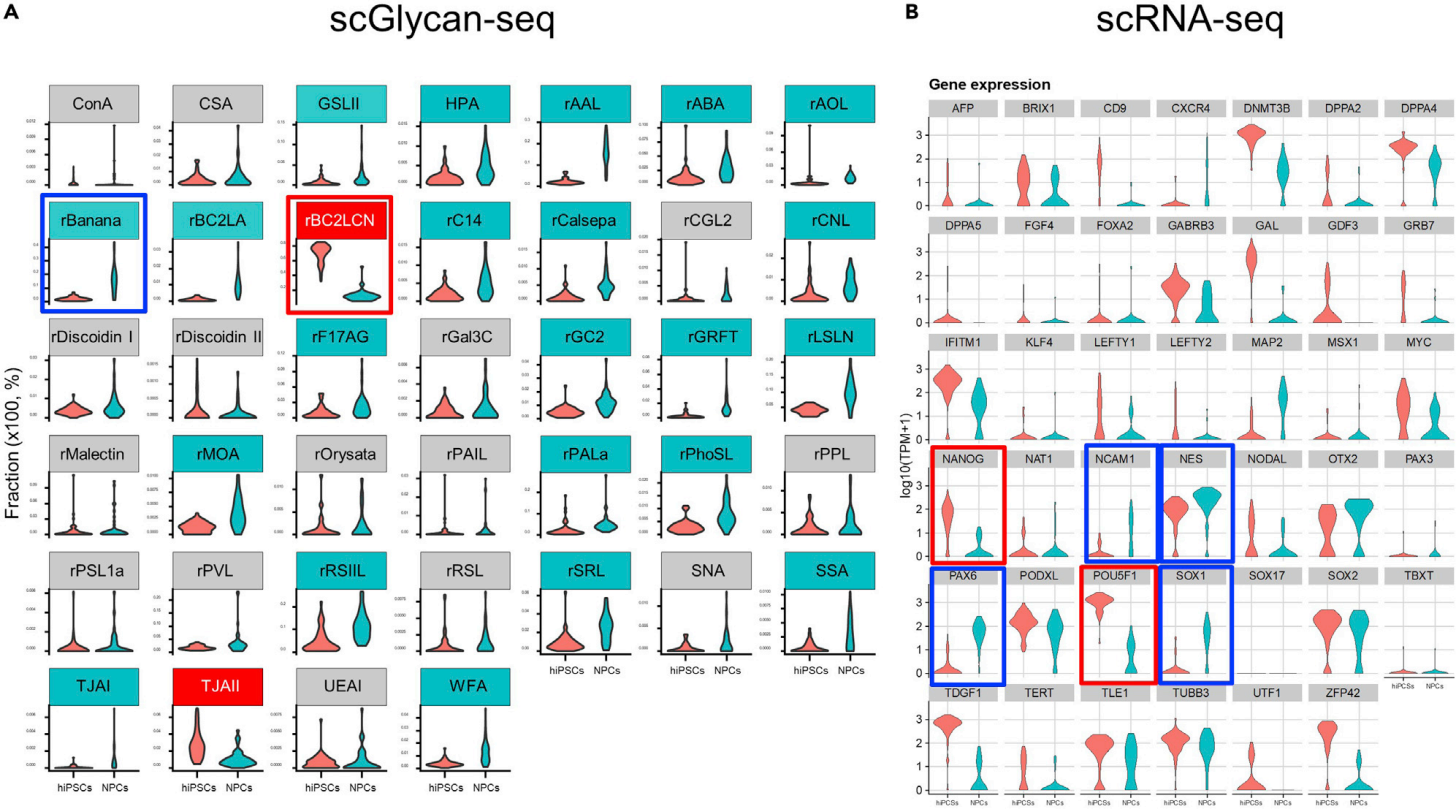
Cell marker expression in scRNA-seq and scGlycan-seq data (A) Violin plots of scGlycan-seq data. (B) Violin plots of 41 selected cell marker gene expression in scRNA-seq data. Red rectangle: iPSC marker, Green Rectangle: NPC marker. Figure reprinted with permission from Minoshima et al. (2021).

A successful scGR-seq output amounts to approximately 5,000 of the total DNA barcode counts. In the case of scRNA-seq, approximately 10,000 genes should be detected. In UMAP, hiPSCs and NPCs are separated into two clusters based on Glycan-seq and RNA-seq data (Figure 3). hiPSC-specific lectin, rBC2LCN, shows higher binding to hiPSCs than NPCs (Figure 4A). In contrast, rBanana shows higher binding to NPCs than hiPSCs (Figure 4A). hiPSCs show higher expression of hiPSC-specific genes such as *NANOG* and *POU5F1* (Figure 4B); in comparison, NPCs show higher expression of NPC marker genes such as NES (NESTIN), PAX6, and SOX1 (Figure 4B).

## Quantification and statistical analysis

### Preprocessing of data

Our in-house developed software, Barcode DNA counting system (Mizuho Information & Research Institute, Inc., Tokyo, Japan), processed the Glycan-seq readout in the FASTQ format, which is accessible from the github (https://github.com/bioinfo-tsukuba/barcode-dna-counting-system). Each read sequence is aligned with the DNA-barcode reference that corresponds to each lectin in this system. Two mismatches in the flanking region and one mismatch in the middle region were accommodated to the maximum extent. As a result, the DNA barcode count data in each cell is a readout. Each lectin count is normalized with the total count of DNA barcode and expressed as % of total count.

***Alternatives:*** Command line interface such as PoolQ might be considered.

Processing of the scRNA-seq readout in the FASTQ format was performed with Subio Platform (version 1.24.5849) according to the manufacturer’s guidelines. Subio Platform can convert FASTQ data into raw count and Transcripts Per Million (TPM) matrix of gene expression, using a pipeline composed of fastp (version 0.20.0), HISAT2 (version 2.1.0), and StringTie (version 2.1.1) having a graphical user interface on both Windows 10 and Mac.

### Quality control

**Figure 5:**
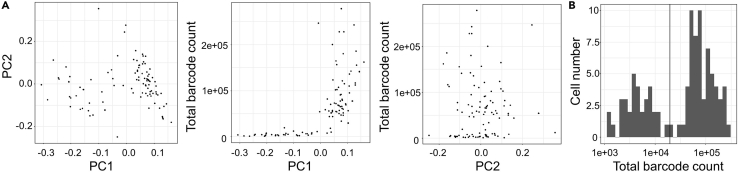
Example of quality control of scGlycan-seq (A) Example of total barcode-biased scGlycan-seq dataset. Principal component analysis (PCA) plot of scGlycan-seq data from 96 fibroblasts (*left*). Scatter plot of component 1 (PC1) or component 2 (PC2) from principal component analysis and total barcode count (*middle*, *right*). Component 1 shows total count-dependent bias. (B) Histogram of total barcode count. Black line indicates cut-off value which is calculated by Otsu’s method using R. Figure reprinted with permission from Minoshima et al. (2021).

Extremely low count data may induce a data bias in scGlycan-seq; it is necessary to check whether the total number of barcode count influence component 1 or component 2 obtained from principal component analysis (Figure 5A). If the total count-dependent bias is detected, then a cut-off value of the total barcode count is determined by Otsu’s method using R with tidyverse packages as follows (Otsu, 1979) (Figure 5B).

1. Function declaration of Otsu’s method

threshold_otsu <- function(x, log10transform=FALSE){

 if(log10transform){

 x_original <- x

 x <- log10(x)

 }

 n <- length(x)

 second_max <- sort(x, partial=n-1)[n-1]

 search_space <- sort(x)[-length(x)]

 sigma <- 0

 th <- 0

 for(th_now in search_space){

 class0 <- x[x <= th_now]

 class1 <- x[x > th_now]

 w0 <- length(class0)

 w1 <- length(class1)

 mu0 <- sum(class0) / w0

 mu1 <- sum(class1) / w1

 sigma_now <- w0 ∗ w1 ∗ (mu0 - mu1)ˆ2

 if(sigma < sigma_now){

 sigma <- sigma_now

 th <- th_now

 }

 }

 if(log10transform){

 th <- x_original[match(th, x)]

 }

 return(list(sigma=sigma, threshold=th))

}

2. Load total count data

df1 <- read_tsv("rawdata.tsv") #Replace rawdata.tsv with your data file name

df1 %>%

 filter(! BarcodeID %in% c("mIgG", "gIgG") ) %>%

 select(- BarcodeID) %>%

 as.matrix() -> mat1

dft <- tibble(Sample_ID = colnames(mat1), Total_count = colSums(mat1))

3. Perform binarization in log10-transformed total count data with Otsu’s method

res_otsu <- threshold_otsu(dft$Total_count, log10transform = TRUE)

print(res_otsu$threshold)

table(dft$Total_count > res_otsu$threshold)

4. Visualization of binarized total count data

n_accepted = sum(dft$Total_count > res_otsu$threshold)

n_rejected = sum(dft$Total_count <= res_otsu$threshold)

dft %>%

 mutate(filtered = Total_count > res_otsu$threshold) %>%

 ggplot(aes(reorder(Sample_ID, Total_count), Total_count)) +

 geom_point(aes(colour = filtered)) +

 geom_hline(yintercept=res_otsu$threshold) +

 theme_bw() +

 scale_colour_discrete(

    name="Filtered",

    breaks=c(TRUE, FALSE),

    labels=c(sprintf("Total count > %d (n=%d)", res_otsu$threshold, n_accepted),

     sprintf("Total count <= %d (n=%d)", res_otsu$threshold, n_rejected))) +

 labs(x="") -> g

plot(g)

In scRNA-seq data, cells with low-quality data are determined by several parameters, such as read count, gene count, or mitochondrial read ratio with Seurat R package (version 4.02). Dead/damaged cells exhibited low read count and increased mitochondrial read ratio whilst aggregated cells showed abnormally high read count. Since an optimum cut-off value to exclude low-quality cells depends on cell type, read depth and read quality, it needs to be determined by individual data set.

### Integrated data analysis

The Seurat R package performs dimensionality reduction, cellular clustering, and identification of differential gene expression and thus can be used to analyze scGlycan-seq and scRNA-seq data. The Seurat platform also supports the integrated analysis of scGlycan-seq and scRNA-seq based on the weighted-nearest neighbor (wnn) workflow. A detailed protocol for the Seurat R package is described in (Hao et al., 2021).

## Limitations

Like flow cytometry and lectin microarray, absolute amounts of glycans and accurate glycan structures cannot be determined directly from the signal intensities described above. Another limitation of the current system is the throughput. Since scGR-seq is a plate-based platform, processing of cell numbers is limited to hundreds of cells, while it can perform full-length total RNA sequencing (Hayashi et al., 2018). In contrast, droplet-based methods such as 10× Genomics (CITE-seq) can sequence thousands of cells at once but target only the 3′ends of poly(A) transcripts (Baran-Gale et al., 2018).

## Troubleshooting

### Problem 1

The molar ratio of DNA barcode relative to lectin is too low (“purification of DNA-barcoded lectins” step 23).

### Potential solution

Increase the amount of PC-DBCO-NHS and incubation time.

### Problem 2

Cells might be partially agglutinated after incubation with DNA-barcoded lectins (“single cell glycan-seq” step 19).

### Potential solution

Cells might be agglutinated during incubation with DNA-barcoded lectins. The cell aggregates can be removed using 100 μm filter. In manual picking, single cells can be selected by visual inspection. In FACS, gating with FSC-H and FSC-W can exclude aggregated cells from the analysis.

### Problem 3

No band is detected when DNA barcodes obtained from each single-cell were run on the microchip electrophoresis system, MultiNA (“single cell glycan-seq” step 28).

### Potential solution

It is recommended to include bulk samples (1 × 10^4^ cells) as a positive control to validate PCR reactions. If the band is detected only in bulk samples, the amount of DNA barcodes obtained from each single-cell is likely too low. Even in that case, it may be detectable in a next-generation sequencer such as Miseq if you follow the protocol in “step 29: Denaturing library DNA if the concentration of library DNA is < 4 nM.”

### Problem 4

Amount of cDNA library obtained from each single-cell is lower than 50–100 fmol (“single cell RNA-seq” step 35).

### Potential solution

Keep an experimental space clean to prevent degradation of RNA by RNase contamination.

Confirm that ethanol is dried out after washing steps of AMPure beads, since the residential ethanol may inhibit the subsequent reactions.

Increase the number of PCR cycle.

Remove low yield samples from sequencing analysis with Next-generation sequencer.

Include whole volume of low yield samples into the mixed cDNA library.

### Problem 5

High amount of primer dimers is detected around 110–130 bp when cDNA library obtained from each single-cell were run on the microchip electrophoresis system, MultiNA (“single cell RNA-seq” step 35).

### Potential solution

It is recommended to remove primer dimers by size-selection of DNA fragments with AMPure XP beads because primer dimers will compete with cDNA to bind flow cell in Next-generation sequencer. The addition of 1.0–1.2 times the volume of AMPure XP to the PCR reaction solution is sufficient to remove 110–130 bp fragments. Note that this selection step may slightly reduce short cDNA fragments around 150 bp.

## Resource availability

### Lead contact

Further information and requests for resources and reagents should be directed to and will be fulfilled by the lead contact, Hiroaki Tateno (h-tateno@aist.go.jp).

### Materials availability

Recombinant lectins are available from FUJIFILM Wako Pure Chemical Corporation or the lead contact upon request.

## Data Availability

The code of the barcode DNA counting system is available from github (https://github.com/bioinfo-tsukuba/barcode-dna-counting-system).

## Supplemental information

Table S1. Primer sequence
